# Micromolecular methods for diagnosis and therapeutic strategy: a case study

**DOI:** 10.18632/oncotarget.25161

**Published:** 2018-04-27

**Authors:** Morad Elbouchtaoui, Iulia Tengher, Catherine Miquel, Charlotte Brugière, Amélie Benbara, Laurent Zelek, Marianne Ziol, Fatiha Bouhidel, Anne Janin, Guilhem Bousquet, Christophe Leboeuf

**Affiliations:** ^1^ Université Paris Diderot, Inserm, UMR_S1165, Paris, France; ^2^ Pathology Department, Hôpital St Louis, APHP, Paris, France; ^3^ Pathology Department, Hôpital Jean Verdier, APHP, Bondy, France; ^4^ Obstetrics and Gynecology Department, Hôpital Jean Verdier, APHP, Bondy, France; ^5^ Université Paris 13, Villetaneuse, France; ^6^ Inserm UMR_S1162, Paris, France; ^7^ Oncology Department, Hôpital Avicenne, APHP, Bobigny, France

**Keywords:** HER2 overexpressing breast cancer, micromethods, cancer therapy, laser-microdissection, trastuzumab-based treatment

## Abstract

An intraductal carcinoma, 55 mm across, was diagnosed on a total mastectomy in a 45-year-old woman. The 2 micro-invasive areas found were too small for reliable immunostainings for estrogen, progesterone, and HER2 receptors. In the sentinel lymph-node, a subcapsular tumor embole of about 50 cancer cells was identified on the extemporaneous cryo-cut section, but not on further sections after paraffin-embedding of the sample.

Considering this tumor metastatic potential, we decided to assess HER2 status on the metastatic embole using pathological and molecular micro-methods. We laser-microdissected the tumor cells, extracted their DNA, and performed droplet-digital-PCR (ddPCR) for HER2 gene copy number variation. The *HER2/RNaseP* allele ratio was 5.2 in the laser-microdissected tumor cells, similar to the 5.3 ratio in the HER2-overexpressing breast cancer cell line BT-474.

We thus optimized the adjuvant treatment of our patient and she received a trastuzumab-based adjuvant chemotherapy.

## INTRODUCTION

HER2 overexpressed breast cancers are accounting for 15 to 20% of breast cancers [1], and strongly benefit from anti-HER2 treatment mainly trastuzumab-based chemotherapies [2, 3].

Robust methods have been developed within the last 20 years to accurately determine HER2 status, including immunohistochemistry (IHC) and fluorescence *in situ* hybridization (FISH). An international consensus updated in 2013 has defined the criteria for HER2 overexpression and/or gene amplification [4]. However, these standard methods have still some limitations, typically small invasive foci in a primary tumor or a lymph node micro-metastasis.

Digital droplet polymerase chain reaction (ddPCR) is a more recent technology that has been tested for the determination of *HER2* gene copy number status in breast and gastric cancers [5–8]. Preliminary studies on large invasive components of breast or gastric cancers have reported an excellent concordance between ddPCR and FISH [5, 6].

It has not been evaluated yet for small invasive foci or micro-metastatic cell clusters.

In this study, we used this methodology in a clinical situation of micro-invasive breast cancer with a lymph-node micrometastasis and an undetermined HER2 status, to optimize the patient's treatment.

## RESULTS

### Micromolecular methods enabled to determine HER2 status of a breast cancer lymph-node micro-metastasis

**Figure 1:**
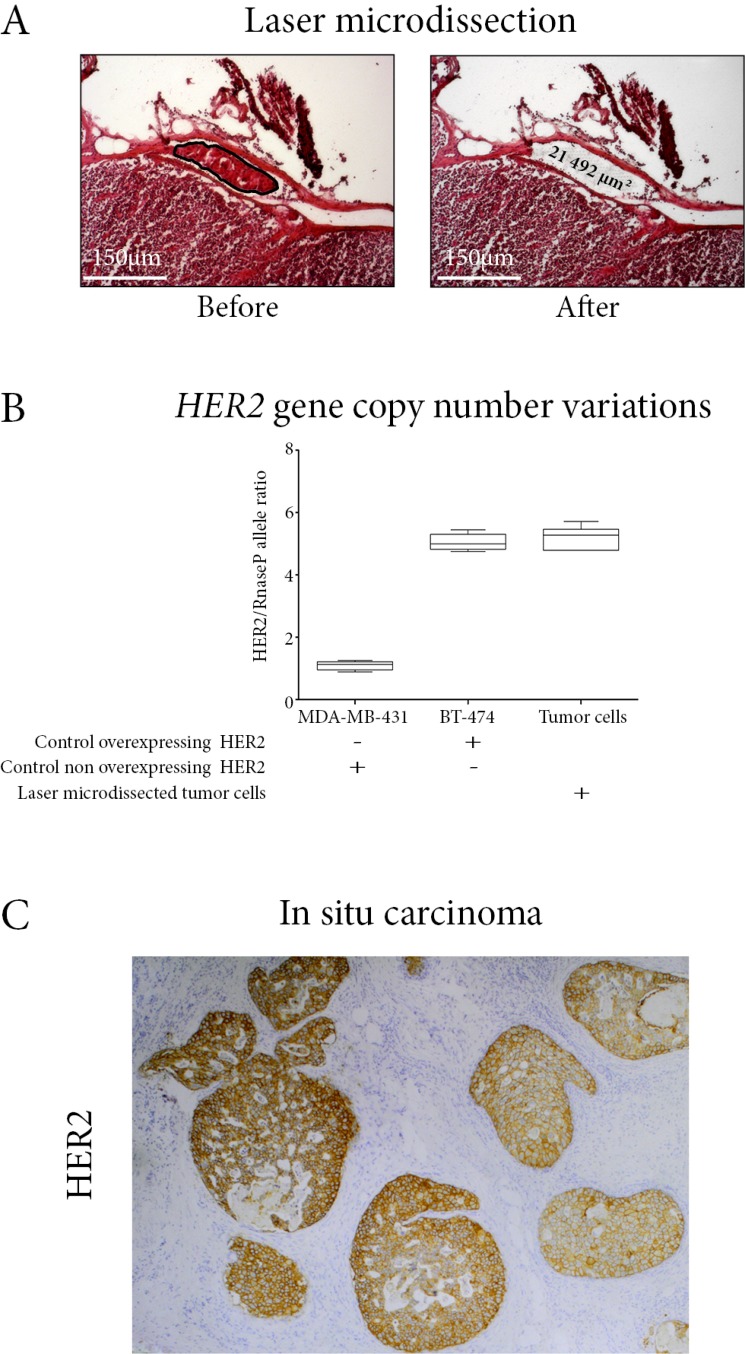
(**A**) Laser microdissection of the intra-lymphatic embole of tumor cells. Lymph node section, hematein-eosin staining. (**B**) HER2 gene copy number of the laser-microdissected tumor cells. Digital droplet PCR method, using BT-474 and MDA-MB-431 cell lines respectively as positive and negative controls. (**C**) HER2 immunostaining on the ductal carcinoma *in situ* component. *In situ* carcinoma cells are overexpressing HER2.

An intra-ductal carcinoma, 55 mm across, was diagnosed on a total mastectomy in a 45-year-old woman. The 2 micro-invasive areas found (<1 mm each) were too small for reliable immunostainings for estrogen, progesterone, and HER2 receptors. In the unique sentinel lymph-node identified using both isotopic and colorimetric methods, a subcapsular cluster of about 50 cohesive cancer cells was identified on the extemporaneous cryo-cut section (Figure 1A), but not on further sections after paraffin-embedding of the sample. The largest diameter of this cancer cell cluster was 220 μm, which defines a micrometastasis (pN1mi) according to UICC international classification [9]. The tumor was thus classified pT1miN1miM0.

Considering this tumor metastatic potential, we decided to assess *HER2* status on the metastatic micrometastasis using pathological and molecular micro-methods. On the 5 μm-thick frozen tissue section of the sentinel lymph node, we laser-microdissected the tumor cells on a total area of 21492 μm^2^, extracted their DNA, and performed droplet-digital-PCR (ddPCR) for *HER2* and *RNAseP* gene copy number variation. We chose *RNAseP* and not *centromere of Ch17 (CEP17)* as reference gene, due to the limited quantity of available material. Contrary to FISH, ddPCR cannot determine the absolute copy number of *HER2*, and a polysomy 17 would have masked a *HER2* amplification.

The *HER2/RNaseP* allele ratio was 5.2 in the laser-microdissected tumor cells, similar to the 5.3 ratio in the HER2-overexpressing breast cancer cell line BT-474 (Figure 1B). HER2 was also overexpressed in the intra-ductal component (Figure 1C). Considering the high metastatic potential (pN1mi), the amplified status of *HER2* with a ratio *HER2/RNaseP* over 5, and the patient's own wish to decrease her relapse risk as far as possible, we decided to perform a trastuzumab-based adjuvant chemotherapy according to national guidelines [10].

### Laser-microdissection combined with ddPCR is a reliable method to determine *HER2* status on small numbers of breast cancer cells

Though ddPCR has not yet been validated by consensus guidelines, several recent studies have shown that ddPCR has excellent correlation with immunohistochemistry or fluorescence *in situ* hybridization for the determination of *HER2* status on large tissue sections of formalin-fixed cancer samples [5, 6]. We aimed to demonstrate that this was also true for small clusters of cancer cells such as micro-invasive foci or micrometastases.

**Figure 2:**
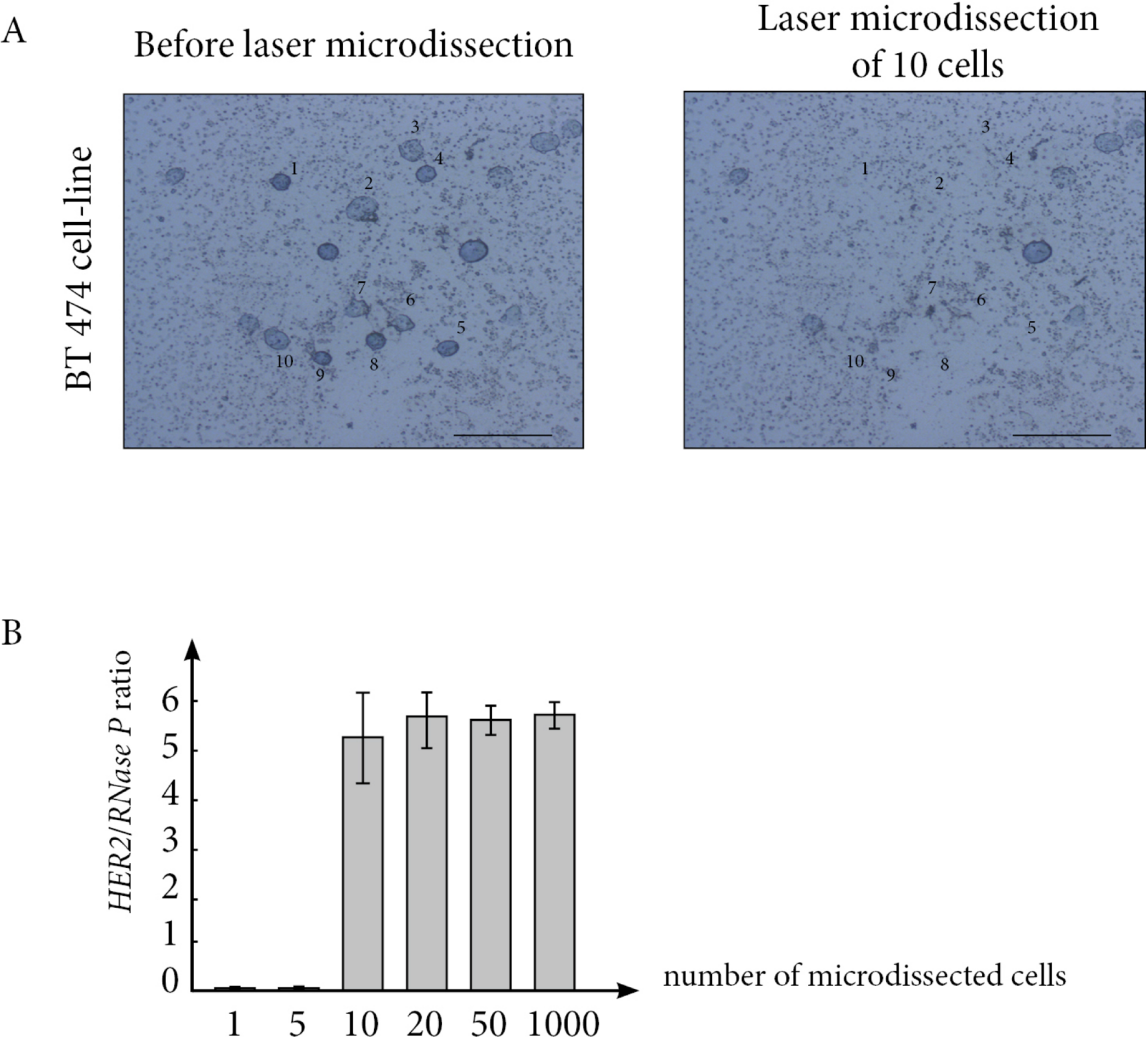
(**A**) Laser-microdissection in liquid medium of BT474 breast cancer cells enables to precisely count and select 10 live cells. (**B**) A minimum of 10 live cells are required to accurately determine *HER2* copy number status.

We first performed a dilution assay with BT-474 HER2-overexpressing cancer cell line. Using laser-microselection to precisely count the total number of cells tested, we assessed *HER2* copy number status on 1, 5, 10, 20 and 50 cells. We demonstrated that 10 cells were sufficient to accurately determine *HER2* copy number status (Figure 2).

Then, we applied our methodology to formalin-fixed tissue sections from biopsies of nine breast cancers with known HER2 status using both immunohistochemistry and FISH. Since we did not have entire cells on tissue sections but only nucleus sections, a reliable result could be obtained only with a minimum of 50 laser-microdissected cancer cells. With this cut-off, we accurately determined *HER2* status compared to immunohistochemistry. Further, in all 9 cases, the range of *HER2* gene amplification was comparable to that determined using FISH (Table 1).

**Table 1:** Comparison of ddPCR and standard methods for assessment of *HER2* status on breast cancer

Patient	IHC HER2 score	FISH		ddPCR	
		HER2 copy number	ratio *HER2*/*CEP17*	ratio *HER2*/*CEP17*	ratio *HER2*/*RNAseP*
Patient 1	0	2.1	**1**	**0.3**	0.4
Patient 2	1	2.2	**1**	**0.8**	0.3
Patient 3	2	3.2	**1.2**	**0.9**	0.4
Patient 4	2	15.1	**1.8**	**1.5**	3
Patient 5	2	5.9	**3**	**4.2**	4.4
Patient 6	2	7.9	**3.9**	**4**	3.3
Patient 7	3	15.4	**5.7**	**9**	5.3
Patient 8	3	21.1	**10.2**	**8.5**	7
Patient 9	3	21.7	**10.3**	**9.4**	12

IHC: immunohistochemistry.

FISH: fluorescence *in situ* hybridization.

ddPCR: digital droplet PCR.

In the case of patient 4 (underlined in grey in Table 1), using FISH, the ratio *HER2/CEP17* was <2 while *HER2* copy number was high, of 15.1. The status of this breast cancer was thus amplified according to 2013 consensus [4]. Using ddPCR, the ratio *HER2/CEP17* was 1.5 because of the polysomy 17 and could not detect *HER2* amplification. By contrast, the ratio *HER2/RNAseP* of 3 was concordant with the amplified *HER2* status.

### Assessment of HER2 status on *in situ* breast cancers with undetermined micro-invasive component

**Figure 3:**
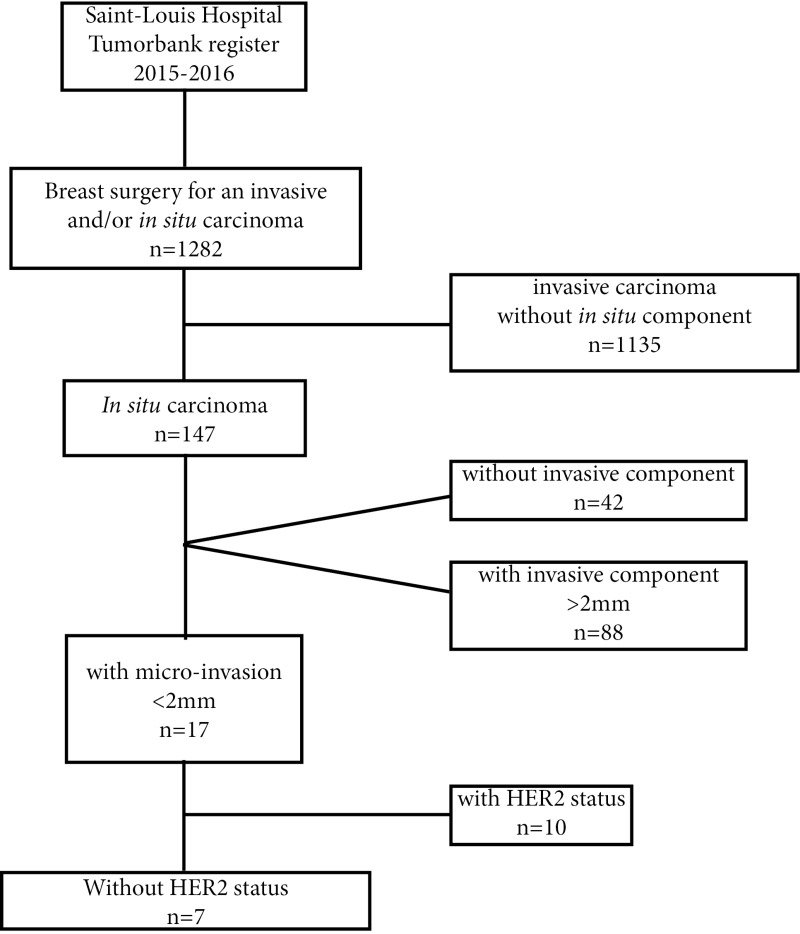
Diagram for selection criteria of the 7 patients with *in situ* carcinoma and micro-invasive component with undetermined HER2 status

We first assessed the frequency of *in situ* breast carcinoma with undetermined micro-invasive component. After interrogation of Saint-Louis hospital tumor bank register from January 2015 to December 2016, we identified 1282 patients with breast surgery for an invasive and/or *in situ* carcinoma, 147 of them with an *in situ* component. When we only retained *in situ* carcinomas with a micro-invasive component (i.e. <2 mm according to international definition), they concerned 17 patients. Seven of them had undetermined HER2 status because of an insufficient quantity of cancer cells with invasive features (5% of cases of *in situ* carcinomas over 2 years in our Cancer Center) (Figure 3).

**Figure 4:**
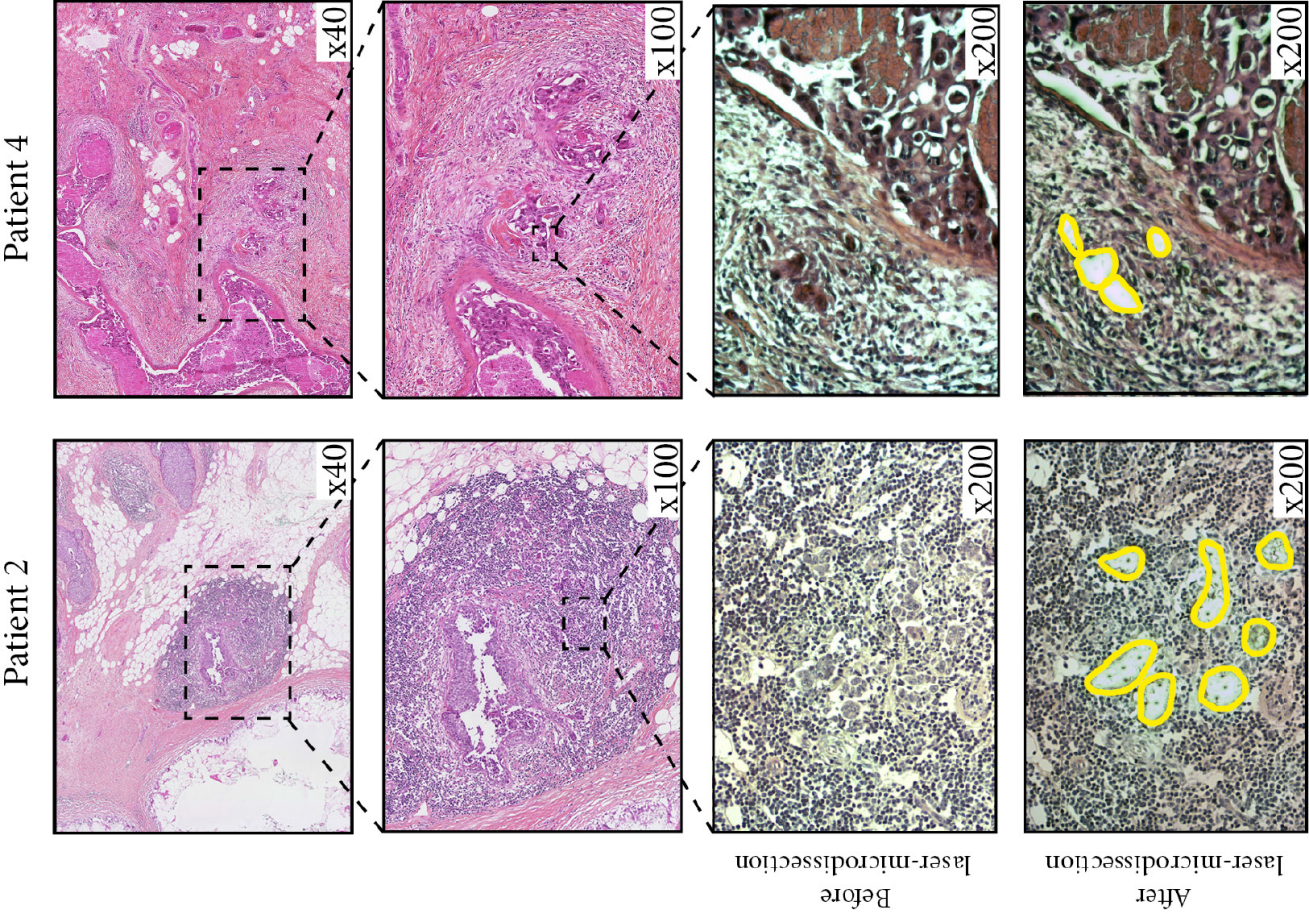
illustrates two cases of patients with *in situ* carcinoma and micro-invasion, for whom we performed laser-microdissection of micro-invasive cells followed by ddPCR, and found an amplified *HER2* status For patient 2 (left panels), hematein-eosin staining of the primary tumor (upper left panels) shows small foci of micro-invasive ductal carcinoma within a strong inflammatory reaction. Laser-microdissection enables the precise selection of these small foci of cancer cells (lower left panels). For patient 4 (right panels), hematein-eosin staining of the primary tumor (upper left panels) shows typical aspect of extensive ductal carcinoma *in situ* surrounded by small clusters of mciroinvasion.

Using our methodology for *HER2* copy number assessment, we were able to determine *HER2* status for 6 of the 7 cases, and *HER2* was amplified in 2 cases (Figure 4 and Table 2). For one case, we could not reach the cut-off of 50 laser-microdissected cells on the micro-invasive component.

**Table 2:** Assessment of *HER2* status on micro-invasive breast cancer using laser-microdissection and ddPCR

Patient	ddPCR
	ratio *HER2*/*RNAseP*
Patient 1	1
**Patient 2**	**5.6**
Patient 3	NE
**Patient 4**	**2**
Patient 5	1
Patient 6	0.7
Patient 7	1

NE: not evaluable.

## DISCUSSION

In the case of our patient, a mere follow-up would have been the rule on the basis of initial clinico-biological data. Tumor cell laser-microdissection on the only sentinel lymph-node section including a lymphatic micrometastasis enabled us to demonstrate *HER2* amplification in these tumor cells, and to optimize the patient's treatment with trastuzumab-based adjuvant chemotherapy in line with international guidelines [10]. In the absence of lymph-node involvement, adjuvant chemotherapy is not fully recommended for HER2-overexpressing breast cancers of less than 5 mm (pT1mi and pT1a) [10, 11]. Here, for decision treatment, we considered two biological characteristics: the high metastatic potential of this micro-invasive primary tumor since the patient already had a lymph node micrometastasis at diagnosis (pN1mi), and the level of *HER2* amplification, since a ratio *HER2/CEP17* over 5 is associated with a higher complete response rate to trastuzumab-based chemotherapy [12]. We finally took into account the patient's own preference to maximally decrease her relapse-risk.

Laser-microdissection enables the selection of cells from solid tumor tissue sections in the same way as flow-cytometry and cell-sorter enable selection from cell suspensions. However laser-microdissection is not fully automatized [13], it produces small quantities of selected cells and micromethods are required for subsequent molecular analyses [14]. ddPCR analyses based on the sample dispersion in droplets, followed by nucleic acid amplification of each droplet, enables detection of molecular events expressed in small numbers of cells. In this study, we evidenced the reliability of our method to accurately determine *HER2* amplification status in 10 live cells and in 50 cancer cells laser-microselected from formalin-fixed tissue sample. This is sufficient in most cases of micro-invasive or micro-metastatic cell clusters.

In the case of our patient with intraductal carcinoma and a single lymph-node micrometastasis identified on the extemporaneous cryo-cut section, we chose to analyse *HER2* copy-number-variation on laser-microdissected cells because, for *HER2*, the level of gene amplification is correlated to protein overexpression [1].

In breast cancer clinical practice, these micromethods of tumor cell dissection and *HER2* molecular analysis extend the limits of tumor invasion characterization for the patients’ benefit. These micromethods could enable therapy optimization for *in situ* cancers with microinvasive areas that are too small for immunohistochemistry analyses. *HER2* gene analyses are particularly suited to laser-microdissected cells because i) the level of gene amplification is correlated to protein overexpression, and ii) trastuzumab targets HER2 overexpressing tumor cells even in micro-invasive and micro-metastatic breast cancers.

## MATERIALS AND METHODS

### Laser-microdissection and DNA processing

A first experimental control used BT474 cells which HER2 copy number is known. For the selection of BT474 cells, living cells were spread on a dedicated slide and laser-microdissected one by one, enabling a precise count of the total number of cells. A total number of 1, 5, 10, 20, 50 and 100 cells were tested. Each experiment was performed in triplicate.

A second *in situ* control used 5 μm-thick formalin-fixed paraffin-embedded tissue sections of breast cancers with known HER2 status. Laser-microdissection was performed to select a precise number of 5, 10, 20, 50, or 100 cells. On tissue sections of breast cancers with micro-invasive component and undetermined HER2 status, the total numbers of invasive cells were counted and a minimum of 50 cells were laser-microdissected. Each experiment was performed in triplicate.

For the patient here studied, laser microdissection was performed on the 5 μm-thick frozen tissue section of the sentinel lymph node, using a Zeiss Microdissection and Pressure Catapulting system (Zeiss, Munich, Germany), to select cancer cells localized within a lymphatic section under the lymph node capsule. The total laser microdissected area was 21492 μm^2^, corresponding to a total of 50 cancer cells.

In all cases, total DNA was extracted from the microdissected cells using DNeasy-Micro-Kit (Qiagen, Courtaboeuf, France), and concentrated in a final volume of 10 μL.

### Droplet-digital PCR method for DNA copy number analysis

For *HER2* gene copy number analyses, total DNA extracted from microdissected tumor cells was used. Total DNA from BT-474 (ductal carcinoma cell line overexpressing HER2) was used as positive control and total DNA from MDA-MB-231 (adenocarcinoma cell line not overexpressing HER2) was used as a negative control. On the 3 different DNA samples, the Droplet Digital Polymerase Chain Reaction (ddPCR) was performed using the QX100 ddPCR workflow system (Biorad, Hercules, CA, USA). The mix contained 20 ng of genomic DNA from microdissected cells, 10 μL of So Fast Eva Green Supermix (Bio Rad), 1 μL of *HER2* probes (Hs00223586_cn, Life Technologies, Foster City, USA) and 1 μL *RnaseP* probes (Taqman^®^ copy number Reference Assay, 4403326, Life Technologies) or 1 μL *Ch17 centromere (CEP17)* probes (see Supplementary File 1) per well, and the final volume for the reaction was 20 μL. Droplets were generated by a QX200 Droplet Generator (Biorad). PCR was carried out on the CFX96 Real Time System (Bio Rad). PCR was performed with an initial denaturing step at 95°C for 10 mn, followed by 40 cycles of denaturing (95°C for 15 s), and annealing (60°C for 1 mn). A post-amplification melting curve program was initiated by heating to 98°C for 10mn and then cooling down to 12°C. Each PCR run included a no-template control. The results of ddPCR were generated using QX100 Droplet Reader (Biorad), and analysed using QuantaSoft software (Biorad). The ratio of *HER2*-positive droplets to *RnaseP*-positive droplets was calculated. A ratio of 0.8–1.2 was considered as a normal copy number for the *HER2* gene.

## SUPPLEMENTARY MATERIALS


