# Supplementary material for: Micromolecular methods for diagnosis and therapeutic strategy: a case study
Source: Oncotarget. 2018 Apr 27;9(32):22862–9. doi: 10.18632/oncotarget.25161 (PMC5978270; doi:10.18632/oncotarget.25161)
Supplement: Supplementary file 1 [file oncotarget-09-22862-s001.pdf]

Micromolecular methods for diagnosis and therapeutic strategy: a case study

SUPPLEMENTARY MATERIALS

Supplementary File 1 : Primer probes for *CEP17*

Forward primer CEP17 : 5′-GCTGATGATCATAAAGCCACAGGTA-3′
Reverse primer CEP17 : 5′-TGGTGCTCAGGCAGTGC-3′
Primer CEP17 : 5′-TGCTGCAATAGGCGG-3′
